# Closing the sea level budget on a regional scale: Trends and variability on the Northwestern European continental shelf

**DOI:** 10.1002/2016GL070750

**Published:** 2016-10-24

**Authors:** Thomas Frederikse, Riccardo Riva, Marcel Kleinherenbrink, Yoshihide Wada, Michiel van den Broeke, Ben Marzeion

**Affiliations:** ^1^ Department of Geoscience and Remote SensingDelft University of TechnologyDelftNetherlands; ^2^ NASA Goddard Institute for Space StudiesNew York CityNew YorkUSA; ^3^ Center for Climate Systems ResearchColumbia UniversityNew York CityNew YorkUSA; ^4^ Department of Physical GeographyUtrecht UniversityUtrechtNetherlands; ^5^ International Institute for Applied Systems AnalysisLaxenburgAustria; ^6^ Institute for Marine and Atmospheric Research UtrechtUtrecht UniversityUtrechtNetherlands; ^7^ Institute of GeographyUniversity of BremenBremenGermany

**Keywords:** sea level budget

## Abstract

Long‐term trends and decadal variability of sea level in the North Sea and along the Norwegian coast have been studied over the period 1958–2014. We model the spatially nonuniform sea level and solid earth response to large‐scale ice melt and terrestrial water storage changes. GPS observations, corrected for the solid earth deformation, are used to estimate vertical land motion. We find a clear correlation between sea level in the North Sea and along the Norwegian coast and open ocean steric variability in the Bay of Biscay and west of Portugal, which is consistent with the presence of wind‐driven coastally trapped waves. The observed nodal cycle is consistent with tidal equilibrium. We are able to explain the observed sea level trend over the period 1958–2014 well within the standard error of the sum of all contributing processes, as well as the large majority of the observed decadal sea level variability.

## Key Points

- Regional sea level budgets at the Northwestern European shelf over the last decades are closed
- Correlation between open ocean steric and shelf sea level is consistent with propagation of coastally trapped waves
- Vertical land motion and solid earth deformation are key to close the budget

## 1 Introduction

Sea level rise is one of the most important consequences of climate change. On regional scales, large deviations from the global mean trend are observed, as well as significant variability on interannual and decadal scales [*Stammer et al.*, 2013; *Hughes and Williams*, 2010]. The emergence of remote sensing techniques and the global Argo program has allowed closure of the sea level budget over the last decade by direct observations of mass and steric components, both on global [*Leuliette and Willis*, 2011] and regional scales [*Rietbroek et al.*, 2016]. However, the presence of multidecadal variability hampers the estimation of long‐term trends from short records. Multiple studies have been undertaken to explain the observed global trends over the past few decades by looking into the sources of sea level change [*Church et al.*, 2011; *Gregory et al.*, 2013; *Hay et al.*, 2015]. On time scales longer than the satellite era, closing the sea level budget on regional scales still forms an open challenge. Knowledge about the origin of trends and variability of regional mean sea level on these longer time scales is of key importance for determining future regional sea level rise, which is needed to ensure coastal safety [*Nicholls and Cazenave*, 2010]. One of the first attempts to close the regional sea level budget on multidecadal time scales has been made by *Slangen et al.* [2014], who studied regional trends, and found an acceptable agreement for most basins, but for individual tide gauge stations, large deviations occur.

In this paper we present a study into the sea level budget at the Northwestern European continental shelf over the period 1958–2014. This region has a dense network of tide gauges, and frequent hydrographic measurements are conducted in the surrounding northeast Atlantic Ocean. Sea level rise in this region is widely recognized [*Wahl et al.*, 2013]. The decadal variability has also been extensively studied and has been linked to wind‐driven coastally trapped waves, which can travel large distances along the shelf edges [*Richter et al.*, 2012; *Calafat et al.*, 2013; *Dangendorf et al.*, 2014]. To date, no studies exist that explain the observed sea level trends in this region. We use a combination of models and observations to determine the influence of mass and steric effects on sea level trends and interannual to decadal variability. GPS observations are used to account for vertical land motion (VLM) that cannot be explained by Glacial Isostatic Adjustment (GIA) and present‐day mass redistribution effects. The modeled sea level is compared to tide gauge observations in the North Sea and along the Norwegian coast.

## 2 Data and Models

**Figure 1:**
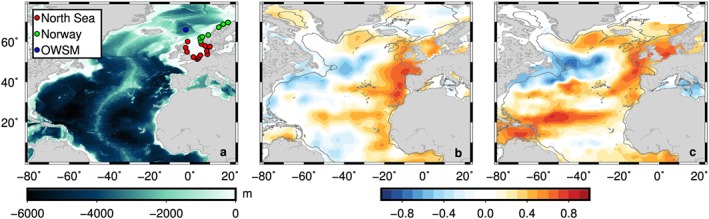
(a) Location of the tide gauges for each region, Ocean Weather Station Mike (OWSM), and bathymetry. (b) Correlation between TG sea level in the North Sea after removing all mass contributors and steric height computed at each grid point from the surface to the seafloor or 1000 m, depending on which is reached first. (c) Correlation between TG sea level in the North Sea and sea level observed by satellite altimetry between 1993 and 2014. The grey line depicts the 1000 m isobath. All time series have been detrended and low‐pass filtered using a 25 month running mean, and all mass contributors have been removed before computing the correlation.

We have obtained monthly tide gauge (TG) records from the Permanent Service for Mean Sea Level (PSMSL) [*Holgate et al.*, 2013]. The tide gauge stations have been divided into two regions, based on their location, as shown in Figure 1a. All stations have a long (50+ years) record, are not subject to known datum instability, and are in proximity of a permanent GPS station. For each station, to reduce the interstation variability, the local effects of wind stress and atmospheric pressure on sea level are removed using simple multiple linear regression with time series from the Twentieth Century Reanalysis Project [*Compo et al.*, 2011], similar to the method of *Dangendorf et al.* [2014]: for each component (sea level pressure anomalies and wind stress in the zonal and meridional direction), the grid point that shows the highest correlation within 250 km around the station is used as regressor. For both regions, an index time series is constructed from the arithmetic mean of all stations that have data at the specific month. Satellite altimetry observations are based on AVISO's multimission gridded sea level anomalies (SLA) product (aviso.altimetry.fr) over 1993–2014. A Gaussian filter with a radius of 150 km has been applied to remove high‐frequency signals.

### 2.1 Mass Signals and Fingerprints

Mass exchange between land and ocean causes a spatial nonuniform relative sea level (RSL) response, due to changes in the geoid and eustatic sea level, and deformation of the solid earth. This effect is taken into account by solving the elastic sea level equation [*Clark and Lingle*, 1977; *Tamisiea et al.*, 2010] including the Earth‐rotational feedback [*Milne and Mitrovica*, 1996].

We consider the mass contribution of glaciers, Greenland and Antarctic ice sheets, and terrestrial water storage (TWS). Glacier mass changes are based on the modeled mass balance of *Marzeion et al.* [2015]. For both ice sheets, a simple input‐output model is used. The surface mass balance (SMB) is modeled by RACMO2.3 [*Noël et al.*, 2015; *van Wessem et al.*, 2014]. For the Greenland ice sheet, estimates for discharge are used as listed in *van den Broeke et al.* [2016]. For Antarctica, output is based on Gravity Recovery and Climate Experiment (GRACE) [*Watkins et al.*, 2015] and the ice sheet mass balance inter‐comparison exercise (IMBIE) [*Shepherd et al.*, 2012] constraints. The mass balance for Antarctica before 1992 is poorly constrained, although semiempirical estimates [*Mengel et al.*, 2016] and polar wander observations [*Mitrovica et al.*, 2015] suggest that the contribution to sea level rise is limited over this period. Therefore, we assume that before 1992, the ice sheet discharge is in long‐term equilibrium with the SMB. Before 1979, when no SMB is available, we assume no mass changes in Antarctica. The spatial partitioning of the mass loss is based on GRACE estimates. The TWS component includes dam retention, natural variability, and groundwater depletion. Dam retention is based on the GRanD database [*Lehner et al.*, 2011] and the method of *Chao et al.* [2008] to determine filling and seepage rates. Natural variability and groundwater depletion are both estimated from the global hydrological model PCR‐GLOBWB [*Wada et al.*, 2010, 2014]. A detailed description of the computation of all mass components, the validation of the ice sheet mass balance, the methods to determine the uncertainties, and the computation of the elastic response is given in the supporting information [*Pfeffer et al.*, 2014; *A et al.*, 2013; *Whitehouse et al.*, 2012; *Rignot et al.*, 2011].

### 2.2 Vertical Land Motion, GIA, and the Nodal Cycle

Tide gauges measure sea level relative to land, and therefore, VLM will affect the observations [*Wöppelmann and Marcos*, 2016]. Both GIA and present‐day mass effects cause solid earth deformation, which results in VLM. To separate these known effects from unknown VLM, we use GPS observations to estimate VLM not explained by GIA and present‐day mass effects:

(1) $$h_{\text{VLM‐r}}(t)=h_{\text{GPS}}(t)-h_{\text{mass}}(t)-h_{\text{GIA}}(t)$$

with *h*_mass_ the solid earth deformation due to large‐scale mass effects and *h*_GIA_ solid earth deformation due to GIA. The residual VLM term *h*_VLM‐r_ hence accounts both for unmodeled VLM and errors in GIA and mass loading models. For each tide gauge station, we determine a linear rate of residual VLM from a nearby permanent GPS site. Processed GPS time series were obtained from Nevada Geodetic Laboratory (geodesy.unr.edu). For the stations along the Norwegian coast, linear trends from *Kierulf et al.* [2014] have been used. Estimates of the GIA impact on VLM and sea level come from the global ICE6G‐VM5a model[*Peltier et al.*, 2015].

The 18.6 year nodal cycle also causes variability on decadal scales. According to a long‐standing belief, the phase and amplitude of the nodal cycle follow the equilibrium law [*Proudman*, 1960; *Woodworth*, 2012]. We account for the nodal cycle by assuming that the amplitude follows the self‐consistent equilibrium law and no phase shift occurs [*Woodworth*, 2012]. The procedure to estimate linear trends and confidence intervals of GPS time series and a figure of the regional GIA estimates can be found in the supporting information [*Bos et al.*, 2013].

### 2.3 Steric Height

The continental shelf is generally shallow, and therefore, the local contribution of steric expansion will be small on interannual time scales. However, steric effects in nearby open ocean will influence sea level on the shelf [*Landerer et al.*, 2007], although serious decoupling between coastal sea level and open ocean steric variability could occur [*Bingham and Hughes*, 2012]. Wind‐driven coastally trapped waves are known to decouple coastal from open ocean sea level and dominate the decadal sea level variability in our region of interest [*Calafat et al.*, 2012, 2013; *Dangendorf et al.*, 2014].

To determine the relationship between open ocean steric signals and shelf sea level, we remove the sea level response to all large‐scale mass contributors and the equilibrium nodal cycle from observed RSL and compute the correlation between the resulting detrended and 25 month low‐pass filtered residual RSL and steric sea level over the North Atlantic. Steric changes have been computed from 3‐D temperature and salinity grids from EN4 version 4.1.1 [*Good et al.*, 2013] from a depth of 1000 m to the surface over the period 1958–2014. The resulting correlation pattern (Figure 1b) shows that the open ocean steric height in the Bay of Biscay and west of Portugal correlates strongly with sea level variability in the North Sea. Satellite altimetry observations show a very similar correlation pattern, and they also point at the coherence between the North Sea and the Norwegian coast (Figure 1c). Therefore, we use the average steric signal over this area as a proxy for the impact of ocean dynamics on the shelf. The area over which the steric height is averaged and the method to compute the time series and accompanying uncertainties are described in the supporting information [*Gouretski and Reseghetti*, 2010; *Pawlowicz et al.*, 2012; *Roemmich and Gilson*, 2009; *Bos et al.*, 2014]. Since open ocean steric anomalies below the shelf bottom affects on‐shelf bottom pressure, self‐attraction and loading effects will amplify the signal. This effect, however, is relatively small [*Richter et al.*, 2013] and, therefore, not taken into account.

### 2.4 Reconstructed RSL

We define our reconstructed RSL as the sum of all contributing processes, which reads

(2) $$\zeta (t)=\zeta_{\text{dyn}}(t)+\zeta_{\text{mass}}(t)+\zeta_{\text{GIA}}(t)+\zeta_{\text{nodal}}(t)-h_{\text{VLM‐r}}(t)$$

*ζ*(*t*) represents reconstructed RSL at time *t*. *ζ*_dyn_ is the dynamic contribution, for which the steric height over the aforementioned area is used, *ζ*_mass_ the local RSL response to the sum of mass effects, and *ζ*_GIA_ the RSL response to GIA. The RSL response to GIA and mass effects consists of geoid and eustatic changes and solid earth deformation. *ζ*_nodal_ the contribution of the nodal cycle, and *h*_VLM‐r_(*t*) the residual vertical land motion as defined in equation (1). The mean has been removed from all time series. Note that the reconstructed RSL is simply the sum of all components and no parameters are estimated in equation (2).

## 3 Results

**Figure 2:**
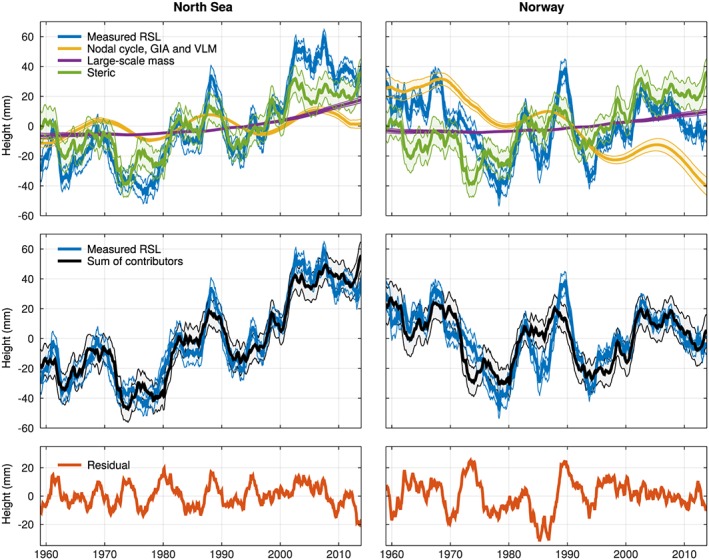
(top) Measured relative sea level, together with the contributing processes, (middle) the sum of the contributing processes, and (bottom) the residual RSL. The shaded areas denote one standard error (sum of contributors) and the spread between the different tide gauge stations (measured RSL). (left column) North Sea stations and (right) Norway stations. All time series have been low‐pass filtered with a 25 month running mean.

The observed and reconstructed relative sea level for both regions of interest is displayed in Figure 2. The dynamic signal shows large variability on decadal scales, while large‐scale mass effects are varying more slowly, but show a clear acceleration. The amplitude of the equilibrium nodal cycle is in the order of 10 mm.

Figure 2 (bottom row) shows that the sum of all contributors explains the large majority of the observed variability on interannual and decadal scales. Along the Norwegian coast, the same dynamic signal is still clearly visible, although the residual shows larger peaks than in the North Sea. After removing the linear trend and applying a 25 month running mean, the fraction of explained variance (*R* ^2^) is 0.79 between the detrended contributors and sea level in the North Sea. The corresponding correlation coefficient is 0.89. For Norway, the numbers are 0.63 and 0.79, respectively.

When we compare the linear trends of the reconstructed and observed sea level (Table 1) we see that the model explains the observed trend well within confidence intervals for both regions. Note that the listed uncertainties are on the 1*σ* level. Significant uplift takes place along the coast, for which a large part is explained by GIA and solid earth deformation due to mass exchange. It must be noted that GIA models predict a steep gradient in vertical land motion and relative sea level along the coastline, which makes the model locally prone to model errors. The residual VLM term can compensate for these errors under the condition that the errors in geoid changes stay small. Without correcting GPS observations for solid earth deformation due to present‐day mass transport, the observed VLM trend over the GPS era would be regarded as a long‐term trend. However, the modeled solid earth response shows an upward acceleration over the period of interest, which causes GPS observations to overestimate the long‐term linear trend in vertical land motion. For the North Sea, removal of solid earth deformation caused by present‐day mass changes results in a decrease of 0.5 mm/yr of the estimated long‐term VLM trend. For Norway, this effect is even larger, due to the proximity of the Greenland ice sheet.

**Table 1:** Linear Trends (mm/yr) in the Individual Processes, Reconstructed and Observed Relative Sea Level for Both Regions, and the Global Mean Over 1958–a

	North Sea	Norway	Global
Glaciers	0.26 ± 0.02	0.09 ± 0.02	0.44 ± 0.05
Greenland	0.00 ± 0.01	−0.04 ± 0.01	0.13 ± 0.02
Antarctica	0.08 ± 0.03	0.08 ± 0.03	0.07 ± 0.03
Dam retention	−0.20 ± 0.03	−0.17 ± 0.03	−0.32 ± 0.05
Groundwater / natural	0.26 ± 0.04	0.27 ± 0.04	0.30 ± 0.05
Dynamic	0.75 ± 0.19	0.75 ± 0.19	0.73 ± 0.11
GIA	0.06 ± 0.02	−0.65 ± 0.16	‐
‐GPS^b^	−0.44 ± 0.10	−2.34 ± 0.18	‐
‐VLM‐r	0.14 ± 0.10	−0.55 ± 0.18	‐
Nodal cycle	0.03 ± 0.00	0.05 ± 0.00	‐
Reconstructed RSL	1.37 ± 0.22	−0.17 ± 0.32	1.35 ± 0.15
Observed RSL	1.38 ± 0.29	−0.23 ± 0.31	

a Global mean dynamic sea level based on steric estimates from *Levitus et al.* [2012] and *Purkey and Johnson* [2010]. All errors represent 1*σ*.

b Not part of the sum of contributors.

Because of the proximity of many glacierized regions and the Greenland Ice Sheet, their contribution is well below the global average, while the dynamic contribution is close to the global mean. Due to the contributions of GIA, vertical land motion, and the nodal cycle, the sum of all contributors to the North Sea is close to the global mean sum of processes. For Norway, no significant upward trend can be found: GIA and residual VLM compensate for all mass and steric effects.

## 4 Discussion

**Figure 3:**
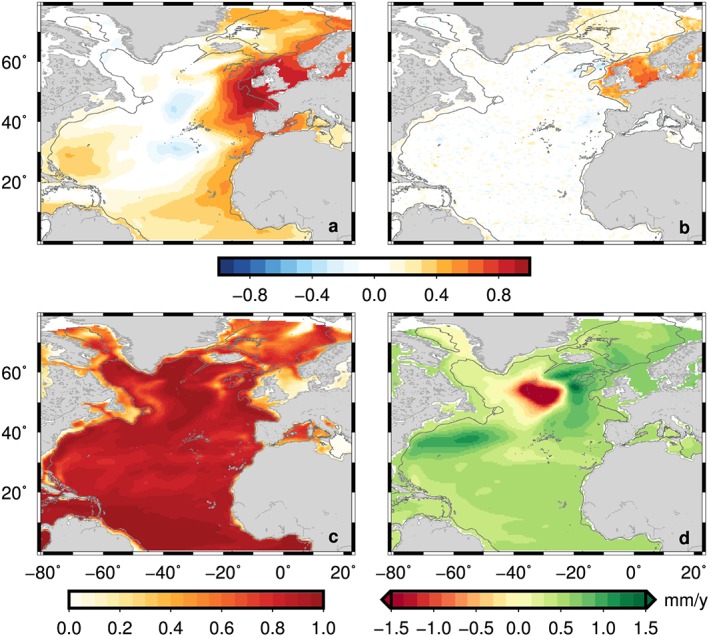
NorESM1‐M (1900–2005) output. (a) Correlation of steric height in the Bay of Biscay with local dynamic sea level. (b) Correlation of steric sea level in Bay of Biscay with local OBP. (c) Fraction of dynamic sea level variability explained by local steric sea level variability. All time series have been detrended and low‐pass filtered using a 25 month running mean. Steric sea levels have been computed from T/S fields up to a depth of 1000 m. The effects of local winds on dynamic sea level have been removed using the same regression model as for the observations. (d) Linear trend in dynamic sea level.

**Figure 4:**
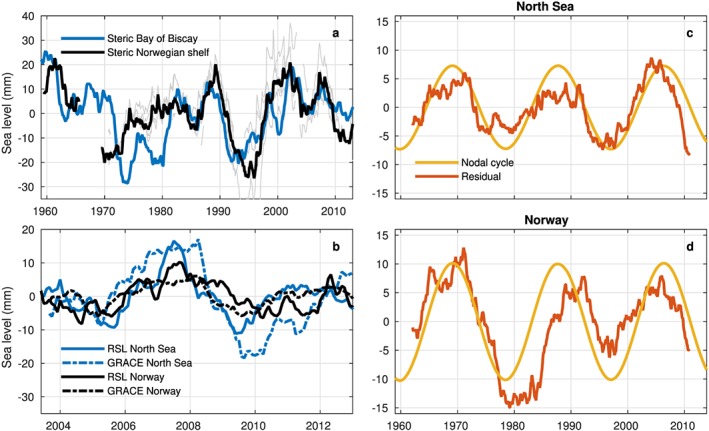
(a) Comparison between observed steric signal in the Bay of Biscay and the steric signal on the Norwegian shelf from hydrographic station measurements. Individual stations in grey. (b) GRACE on‐shelf mass signals and RSL for both regions. All time series in Figures 4a and 4b have been detrended and low‐pass filtered using a 25 month running mean. (c and d) Comparison of measured RSL after subtraction of all terms in equation (2), except for the nodal term versus the equilibrium nodal cycle. The residual time series have been detrended and low‐pass filtered using a 97 month running mean.

To evaluate the properties of the correlation pattern between the open ocean steric and along‐shelf sea level signal, we use the historical run from Fifth Coupled Model Intercomparison Project (CMIP5) Earth system model NorESM1‐M [*Bentsen et al.*, 2013]. The ocean component has a horizontal resolution of 1° and the isopycnal‐coordinate model is mass conserving, which makes the model suitable to study sea level variability driven by changes in steric height and ocean bottom pressure [*Richter et al.*, 2013]. The model is able to reproduce the observed longshore coherence, as depicted in Figure 3a. Figure 3b shows that the open ocean steric signal correlates with the mass signal in the North Sea and at the Norwegian shelf. Along the Norwegian coast, local steric effects still explain the majority of sea level variability (Figure 3c). Consequently, to maintain longshore coherence, the steric component along the shelf should mimic the steric effect in the Bay of Biscay. Steric heights computed from T/S profiles from fixed hydrographic stations along the Norwegian coast (Bud, Sognesjøen, Ytre Utsira, Indre Utsira, and Lista, data from imr.no/forskning/forskningsdata/stasjoner, a location map can be found in the supporting information) confirm the coherence between open ocean and on‐shelf steric variability on the lowest frequencies (Figure 4a), though on subdecadal scales, differences can be noticed. GRACE‐derived on‐shelf Ocean bottom pressure (OBP) signals (Figure 4b; see in supporting information *Klinger et al.*, 2016; *Klees et al.*, 2008; *Swenson et al.*, 2008; *Cheng et al.*, 2013; *Dobslaw et al.*, 2013 for details) are consistent with sea level on both shelves, which indicates that the remote steric signal also appears as an on‐shelf mass signal. The observed along‐shelf coherence corresponds well with the presence of coastally trapped waves, which travel counterclockwise on the Northern Hemisphere [*Huthnance*, 1978]. These waves have the ability to create a spatially coherent monthly to decadal sea level signal over along‐shelf distances exceeding thousand kilometers [*Hughes and Meredith*, 2006; *Sturges and Douglas*, 2011]. Along the eastern Atlantic boundary, longshore winds drive coastally trapped waves over long distances, leading to a coherent decadal sea level signal [*Calafat et al.*, 2012, 2013]. These waves trigger westward traveling Rossby waves, causing open ocean adjustment to coastal sea level [*Marcos et al.*, 2013]. Hence, the open ocean and shelf sea are both affected by decadal variability in the longshore winds, which explains the observed correlation pattern. The propagation of the coastal signals into the open ocean is hampered by decreasing Rossby wave speeds at high latitudes, and hence, the correlation between shelf sea level and the nearby open ocean steric height vanishes at high latitudes.

The coastally trapped waves will be continuously affected by regional longshore winds when propagating northward, thereby gradually altering the wave properties, which may be one of the causes of the residual signal for the Norwegian stations in Figure 2 and corresponds well to the findings of *Calafat et al.* [2013], who show that Norwegian coastal sea level is affected by regional and remote longshore winds.

The steric signal in the Bay of Biscay is also used as a proxy for the long‐term trend in dynamic shelf sea level, with the underlying assumption that the trend does not show large variations over our region of interest. Since the long‐term trend is likely to be driven by other processes than longshore wind variability, this assumption must be verified. The composite steric height time series from the permanent hydrographic stations along the Norwegian coast has a linear trend of 0.68 ± 0.26 mm/yr. Ocean Weather Station Mike, located in the interior of the Norwegian Sea (Figure 1a), shows a steric height trend of 0.72 ± 0.19 mm/yr over the upper 1000 m between 1958 and 2009. Both the shelf and open ocean trends are close to the observed 0.75 ± 0.19 mm/yr in the Bay of Biscay. The dynamic sea level trends in NorESM (Figure 3d) also depict the low spatial variability of dynamic sea level trends in the region.

Some studies suggest that the observed nodal cycle departs from astronomical equilibrium [*Houston and Dean*, 2011; *Baart et al.*, 2011] and explains a large fraction of the observed decadal variability. We have redone our analysis, but without the nodal term in equation (2). The resulting residual (Figures 4c and 4d) shows a phase and amplitude who are in agreement with equilibrium tide. This observation corresponds well to the conclusion of *Proudman* [1960] that the sea level response to the nodal tide should follow the equilibrium law.

## 5 Conclusions

Sea level trends and decadal variability over the North European continental shelf over the second half of the twentieth century until 2014 have been studied. The combination of mass, steric, and solid earth deformation effects explains the large majority of interannual and decadal variability in the region as well as the observed trends. The trend varies throughout the region due to GIA and unmodeled VLM. GPS measurements can partially compensate uncertainties in modeled GIA. A strong correlation is found between steric variability in the open ocean in the Bay of Biscay and west of Portugal and sea level on the shelf. This correlation is not only visible in hydrographic observations but also in satellite altimetry and an Earth system model and is consistent with the existence of wind‐driven coastally trapped waves that radiate westward traveling Rossby waves into the open ocean. The linear trend in dynamic sea level is consistent over the region, and the observed steric height in the Bay of Biscay and west of Portugal is used as a proxy for the trend and decadal variability in dynamic shelf sea level. This proxy explains the vast majority of the observed decadal sea level variability, while the mass contributors mostly explain longer‐term changes. The observed nodal cycle, for which equilibrium tidal theory predicts a relatively large amplitude in the region of interest, follows the equilibrium law. The steric signal shows large variability at decadal time scales, which contaminate nodal cycle estimates from classical least squares. Estimating the nodal cycle from tidal equilibrium, as proposed by *Woodworth* [2012], is therefore the preferred method.

The North Sea shows an upward trend, while along the Norwegian coast, GIA and VLM offset the mass and dynamic effects, which leads to a negligible trend over the studied period. The method used in this study is easily applicable to other regions as long as high quality regional measurements of RSL, VLM, and T/S profiles are available and could provide more insight in the link between coastal and open ocean sea level changes and help improving future coastal sea level forecasts.

## Supporting information



Supporting Information S1
