# Supplementary material for: Closing the sea level budget on a regional scale: Trends and variability on the Northwestern European continental shelf
Source: Geophys Res Lett. 2016 Oct 24;43(20):10864–72. doi: 10.1002/2016GL070750 (PMC5302015; doi:10.1002/2016GL070750)
Supplement: Supplementary file 1 — Supporting Information S1 [file GRL-43-10,864-s001.pdf]

Supporting Information for "Closing the sea level budget on a regional scale: trends and variability on the Northwestern European continental shelf"

Thomas Frederikse[1], Riccardo Riva[1], Marcel Kleinherenbrink [1], Yoshihide Wada[2,3,4,5], Michiel van den Broeke[6], Ben Marzeion [7]

[1]Department of Geoscience and Remote Sensing, Delft University of Technology, The Netherlands
[2]NASA Goddard Institute for Space Studies, New York City, New York, USA
[3]Center for Climate Systems Research,Columbia University, New York City, New York, USA
[4]Department of Physical Geography, Utrecht University, Utrecht, The Netherlands
[5]International Institute for Applied Systems Analysis, Laxenburg, Austria
[6]Institute for Marine and Atmospheric Research Utrecht, Utrecht University, The Netherlands
[7]Institute of Geography, University of Bremen, Bremen, Germany

Contents

1. Text S1 to S3
2. Figures S1 to S5
3. Table S1

Text S1: GPS post-processing

Trends

For vertical land motion at tide gauges, horizontal and vertical positions in the IGS08 reference frame have been obtained from the Nevada Geodetic Laboratory (http://geodesy.unr.edu), together with epochs of known equipment changes. Vertical trends are obtained following a step-wise procedure:

1. For the time series of north, east, and vertical positions, estimate a provisional trend by fitting a linear trend, offsets at times with known equipment changes or data gaps, an annual cycle and a semi-annual cycle.
2. Subtract the estimated model from the time series and remove outliers (larger than 3σ) in the residual.
3. Following the JPL strategy, detect possible offsets in the residual computed at 1 and 2 in all directions by determining the χ^2-statistic for each point with and without break:

$$F(t) = \frac{\chi^2_{\text{no break}} - \chi^2_{\text{break}}}{\chi^2_{\text{break}}} \cdot \frac{n-4}{2} \tag{1}$$

   χ^2 is estimated from the sum of squared residuals. n is the number of data points. A point with $F > 25$ is a possible offset candidate. The possible offset is subsequently removed from the provisional time series. This procedure is iterated until no new possible offset candidates are found.
4. Estimate the size of the vertical offset for all offset candidates (equipment changes, data gaps and detected offset candidates). For jumps not related to equipment changes, only take offsets with a minimum of 4 mm into account.
5. The solid earth deformation from all mass-transport effects is estimated using the sea-level equation, and subtracted from the GPS time series. The solid earth deformation due to GIA is also removed. A linear trend and confidence interval is estimated from the corrected time series using Hector software (*Bos et al.* [2013], segal.ubi.pt/hector).

Corresponding author: Thomas Frederikse, `t.frederikse@tudelft.nl`

> The time series is assumed to consist of a linear trend, an annual and semi-annual cycle. The noise is assumed to follow a combined white and power law distribution.

For Norwegian tide gauges, no GPS data is included in the Nevada Geodetic Lab database. Therefore, we make use of the estimates of *Kierulf et al.* [2014]. Solid earth deformation effects are removed from the trend. The GPS uncertainty is assumed to stay constant over the whole time span:

$$\sigma_t = \sigma_{\text{trend}} \, |t - t_0| \tag{2}$$

t_0 is the mean time of the GPS time series. For the Norwegian GPS stations we use the provided uncertainties from *Kierulf et al.* [2014], which are based on a Flicker noise model. All GPS trends are assumed to be independent, so the regional VLM uncertainty is the square root of the quadrature sum of the individual errors.

Text S2: Contributors and fingerprints

Sea level response to mass transport

To determine the effect of large-scale sea level changes due to mass transport in the earth system, we compute fingerprints that use mass gain or mass loss on a specific location to calculate the response of the geoid, solid earth and relative sea level on a 0.5° grid. Since the time scales of the mass transport terms in this study are decadal in origin, we only regard the elastic response. A local change of load $\Delta L(\theta, \phi)$ results in three distinct effects:

1. A change in the geoid height: $\Delta G(\theta, \phi)$
2. A change in the solid earth height : $\Delta C(\theta, \phi)$
3. A change in relative sea level: $\Delta R(\theta, \phi) = \Delta G(\theta, \phi) - \Delta C(\theta, \phi) + \mathcal{L}$

$\mathcal{L}$ is the global-mean sea level change due to requirement of mass conservation. To obtain the resulting changes in geoid, solid earth and RSL, we solve the sea level equation [*Clark*, 1977] by means of the pseudo-spectral method, as described by *Tamisiea et al.* [2010], together with the earth rotational feedback [*Milne and Mitrovica*, 1996]. To prevent aliasing effects, a Gaussian filter is applied to all input loads with a radius of 42 km. We use a center-of-mass reference frame, and compute our solution up to spherical harmonic degree 360.

Glaciers

Mass changes due to glacier melting are based on *Marzeion et al.* [2015]. The regional glacier mass balances of all individual regions are used, except for Greenland and Antarctica, since they are included in the mass change signal of the ice sheets. The glacier mass balance for 2014 is estimated using simple linear extrapolation. Each regional mass balance estimate is mapped onto the corresponding glacier region mask from Randolph Glacier Inventory 5.0 [*Pfeffer et al.*, 2014]. For each region, the sea level equation is solved, and all individual regions are added for the total sea level response. From the regional mass balance error estimates, the upper and lower bound for each region is calculated. The sea level equation is also solved for the upper and lower bound mass estimates, and the absolute values of the fingerprint difference with the mean for each region are added in quadrature to obtain the mean glacier error estimate. To obtain estimates of uncertainties in the trend, a linear trend is also estimated from the fingerprints resulting from the upper bound of the mass estimate. The difference between the two trends is used as an estimate of the trend uncertainty.

Ice sheets

The contribution of ice sheets is estimated using an input-output method (IOM). The results have been calibrated by the estimates from the IMBIE intercomparison project [*Shepherd et al.*, 2012] and GRACE data. For GRACE, the JPL 3° by 3° mascon solution is used,

together with a Coastline Resolution Improvement scheme (*Watkins et al.* [2015], data downloaded from dx.doi.org/10.5067/TEMSC-OCL05). For Greenland, the GIA correction of *A et al.* [2013], and for Antarctica, the V2a model from *Whitehouse et al.* [2012] is applied.

The input comes from RACMO2.3 [*Noël et al.*, 2015; *van Wessem et al.*, 2014] surface mass balance (SMB), for the period 1958-2014 for Greenland and 1979-2014 for Antarctica. For Greenland, the output estimate for 1991-2014 is based on results listed in *van den Broeke et al.* [2016], which estimates an acceleration of 6.6 Gt/y^2 over this period. Before 1991, we assume that the ice sheet is in long-term balance: we estimate a linear trend through the total SMB between 1958 and 1991, and use this trend as an estimate for the ice discharge before 1991.

For Antarctica, *Rignot et al.* [2011] estimates an ice discharge acceleration of 9.0 Gt/y^2 since 1992. When this estimate is used for Antarctica, the resulting mass imbalance is larger than IMBIE and GRACE estimates. Therefore, we use a heuristic value of 3.0 Gt/y^2 for the acceleration of the discharge, which gives a good fit to both IMBIE and GRACE. For the period 1979-1992, we assume long-term balance, similar to Greenland. Before 1979, we assume that the ice sheet is in balance (SMB-discharge = 0).

The load is not uniformly applied to the whole ice sheet, but partitioned following the GRACE mascon estimates, for which a linear trend in each 3° by 3° mascon block is calculated.

Since RACMO2.3 does not provide uncertainty estimates, we assume that the error grows with the average error accumulation of IMBIE. The resulting total mass balance, together with the validation time series is depicted in figure 1. Similar to the glacier mass balance, the uncertainty in the resulting regional sea level trends and variability is estimated from fingerprints of the upper bound of the mass balance.

Terrestrial water storage

In the model, three TWS effects are included: dam retention, groundwater depletion and natural variability. For dam retention, we use the method of *Chao et al.* [2008] to determine the filling and seepage rate. The dam data has been obtained from the GRanD Global Reservoir and Dam database [*Lehner et al.*, 2011], and the load is attributed to the nearest grid cell. The list contains 6862 dams. Dams from the Netherlands all enclose former seas, which only cuases mass effects if the lake water level deviates from mean sea level. We assume that this deviation is small and therefore, these dams have been removed from the list.

The effects of natural hydrology are estimated using PCR-GLOBWB version 2.0 [*Wada et al.*, 2014], run on a 0.5° horizontal resolution. For groundwater depletion, the estimates of *Wada et al.* [2010] have been used. For all processes, we assume a rate uncertainty of 15 %.

Nodal cycle

For the nodal cycle, we use the principle of *Proudman* [1960], which states that the nodal tide should almost certainly follow the equilibrium law, which reads:

$$\Omega = \alpha \cdot (1 + k_2 - h_2) \cdot (3 \sin^2 \theta - 1) \tag{3}$$

with tidal love numbers $k_2 = 0.36$ and $h_2 = 0.60$, tidal magnitude $\alpha = 8.8$ mm and latitude θ. Following *Woodworth* [2012], we calculate the self-attraction and loading response of this tidal load and add the resulting changes in geoid, crust and RSL to the equilibrium sea level response.

Steric height

The EN4 objectively analyzed gridded product has been used [*Good et al.*, 2013], with the XBT and MBT bias corrections of *Gouretski and Reseghetti* [2010] applied. From the T and S fields, a steric height is calculated from a depth of 1000 m to the surface using the TEOS-10 software package (*Pawlowicz et al.* [2012], www.teos-10.org). To determine the relationship between the tide gauge sea level and the steric effect, two products are computed:

1. A spatial grid with the correlation between de-trended sea level, corrected for all but the steric term and the gridded detrended steric sea level. Both time series are low-pass filtered using a 25-month running mean.
2. The same grid as above, but the R^2-value instead of the correlation.

The points with a correlation above 0.5 and a positive value for R^2 are selected and used. Figure 3 shows the grid points, over which the average steric component has been calculated.

For each grid point, EN4 provides a standard error for the salinity and temperature. We use a Monte-Carlo simulation by, for each month, perturbing the mean fields with a normally distributed error with the provided standard deviation. We simulate 100 realizations per grid cell. The perturbation is equal for all depth levels as it is reasonable to assume that the errors are highly correlated in the vertical direction. The standard deviation in the produced steric height is then used as a standard error of the steric height at each grid cell. We then calculate the mean and standard standard error for the whole region by using the following spatial covariance function, based on *Roemmich and Gilson* [2009]:

$$\sigma_{ij} = \sigma_i \sigma_j \left[0.77 \mathrm{e}^{-\left(\frac{\alpha l}{140}\right)^2} + 0.23 \mathrm{e}^{-\frac{\alpha l}{1111}} \right] \tag{4}$$

l is the distance between point i and j. When calculating the running mean, the monthly steric errors are assumed to be independent. Variability in the tide gauge records mostly shows generalized Gauss-Markov (GGM) distribution of temporal correlated noise [*Bos et al.*, 2014]. Since the tide gauge variability mainly origins from steric variability, we also use GGM as model for the temporal correlation of the noise in the steric component. The linear trends are computed using Hector software.

Text S3: GRACE estimates

The monthly GRACE OBP time series are estimated using post-processed ITSG-Grace2016 time-varing gravity fields [*Klinger et al.*, 2016], averaged over the polygons shown in figure 4. These gravity fields are provided with full variance-covariance matrices, which allows the use of an anisotropic Wiener filter [*Klees et al.*, 2008] to reduce the noise and striping effects. ITSG monthly gravity solutions are computed with respect to a static gravity field including a secular trend and an annual cycle and the Atmosphere and Ocean Dealiasing (AOD1b) product [*Dobslaw et al.*, 2013]. The anisotropic Wiener filter is applied to the solutions after which the annual and secular signal are restored. Geocenter motion (degree 1) is estimated for the ITSG gravity fields based on the method of *Swenson et al.* [2008]. The C20 component is replaced by satellite laser ranging estimates from *Cheng et al.* [2013]. Eventually, the monthly averages of the OBP component of the AOD1b product are restored.

Figure S1

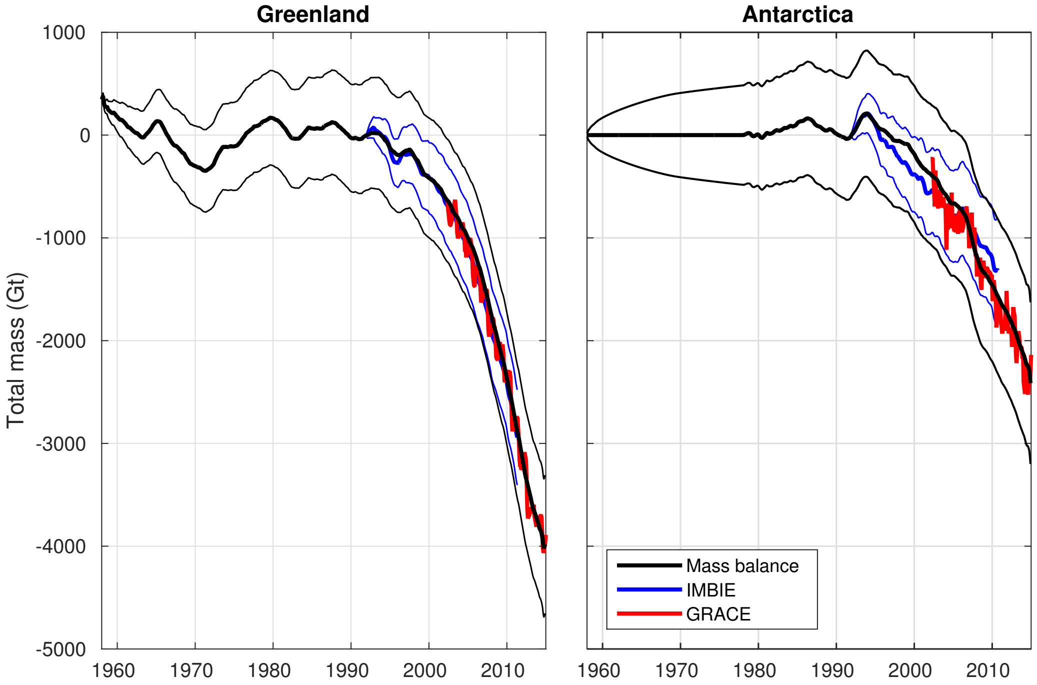

Figure 1. Estimate of Greenland and Antarctic mass balance, compared with IMBIE and GRACE estimates

Figure S2

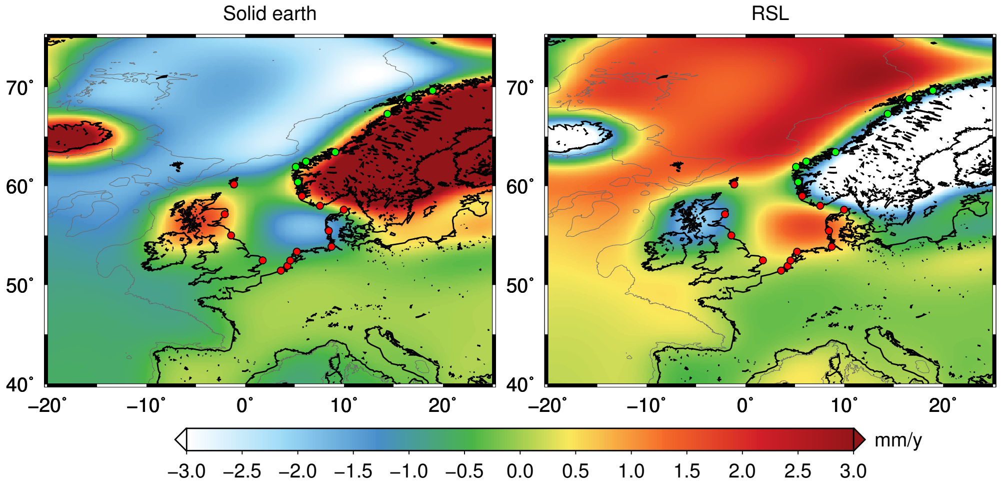

Figure 2. Present-day vertical crustal motion (left) and relative sea level (right) due to GIA from a global model (ICE-6G VM2a, *Peltier et al.* [2015]).

Figure S3

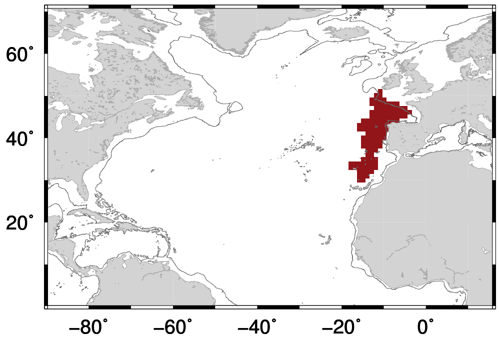

Figure 3. Grid points for which the EN4 steric height has a coefficient of determination $R^2 > 0.0$ and a correlation > 0.5, with North Sea sea level after removal of known mass effects. These points are averaged to form the deep sea steric signal

Figure S4

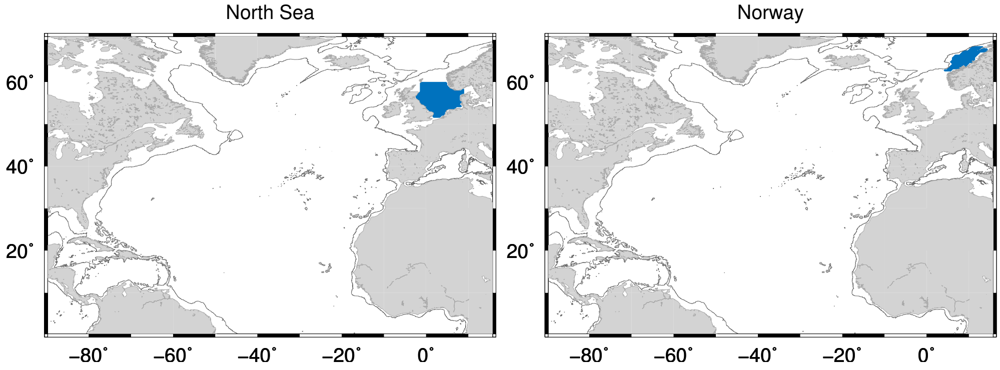

Figure 4. Averaging regions for the GRACE-derived mass signals in the North Sea and along the Norwegian coast

Figure S5

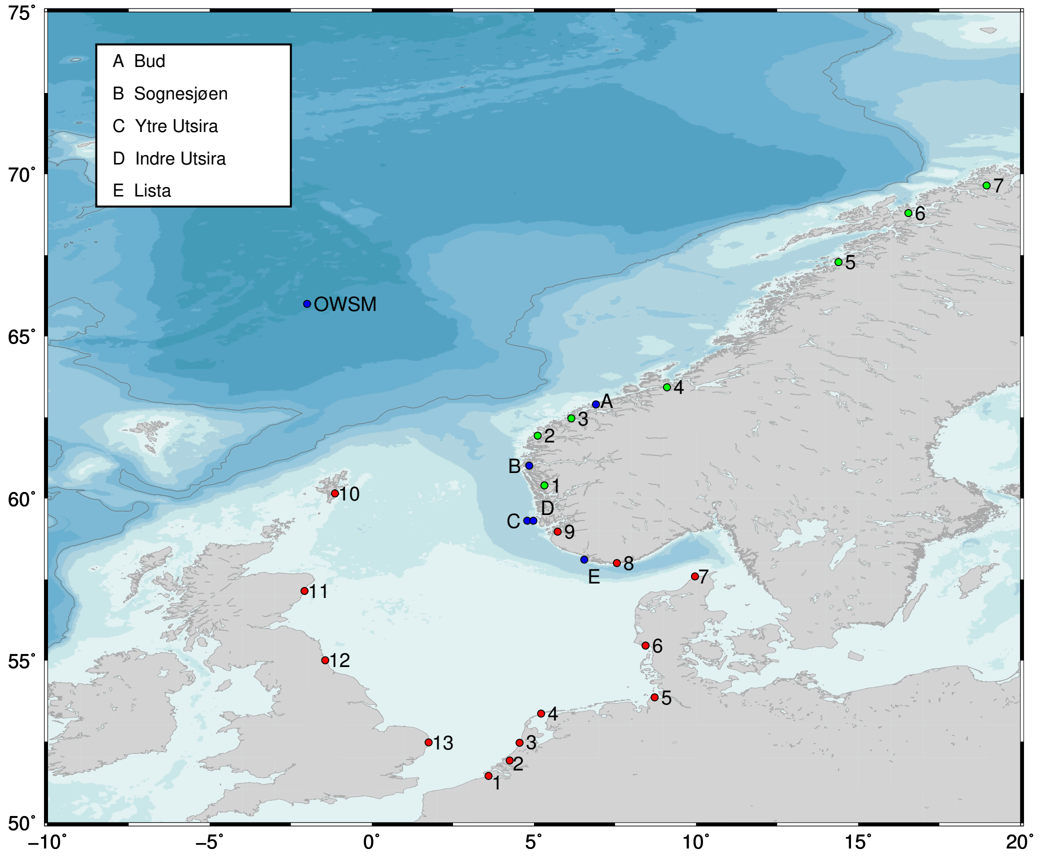

Figure 5. Locations of the tide gauge stations, Ocean Weather Ship Mike (OWSM) and fixed hydrographic stations. The tide gauge labels correspond to the tables S1.

Table S1: TG and GPS stations

Table 1. Tide gauge stations and accompanying GPS stations for the North Sea (NS) and the Norwegian coast (NS). Numbers correspond to figure S5. Linear GPS rates (h_{GPS}), GPS corrected for crustal deviation from load effects and GIA($h_{\text{VLM-r}}$). Sum of all processes at each individual station and observed sea level trend. All rates in mm/y. All errors are on the 1σ level.

		Tide gauge	GPS	h_{GPS}	$h_{\text{VLM-r}}$	Sum	Observed
1	NS	Vlissingen	VLIS	-0.27 ± 0.38	-0.80 ± 0.38	2.15 ± 0.43	1.94 ± 0.30
2	NS	Maassluis	DELF	-0.73 ± 0.26	-1.01 ± 0.26	2.43 ± 0.33	2.04 ± 0.25
3	NS	IJmuiden	WIJK	-0.51 ± 0.33	-0.88 ± 0.33	2.48 ± 0.39	2.02 ± 0.33
4	NS	W-Terschelling	TERS	-0.64 ± 0.29	-0.43 ± 0.29	2.35 ± 0.39	1.78 ± 0.37
5	NS	Cuxhaven	TGCU	0.11 ± 0.52	0.13 ± 0.52	1.80 ± 0.59	1.73 ± 0.50
6	NS	Esbjerg	ESBC	0.43 ± 0.31	1.10 ± 0.31	1.53 ± 0.52	1.80 ± 0.40
7	NS	Hirtshals	HIRS	2.40 ± 0.31	1.11 ± 0.31	-0.29 ± 0.39	0.24 ± 0.30
8	NS	Tregde	TGDE	1.99 ± 0.15	0.89 ± 0.15	0.09 ± 0.26	0.27 ± 0.22
9	NS	Stavanger	STAS	1.37 ± 0.57	-0.20 ± 0.57	0.83 ± 0.63	0.66 ± 0.26
10	NS	Lerwick	LWTG	0.71 ± 0.57	-0.06 ± 0.57	1.40 ± 0.61	0.11 ± 0.44
11	NS	Aberdeen	ABER	0.89 ± 0.12	-0.83 ± 0.12	0.77 ± 0.46	1.26 ± 0.38
12	NS	North Shields	NSTG	0.96 ± 0.21	-0.03 ± 0.21	0.70 ± 0.33	1.57 ± 0.33
13	NS	Lowestoft	LOWE	-0.44 ± 0.15	-0.49 ± 0.15	2.06 ± 0.26	2.59 ± 0.38
1	NO	Bergen	BRGS	1.50 ± 0.32	-0.54 ± 0.32	0.59 ± 0.48	0.54 ± 0.25
2	NO	Maløy	FLOC	1.40 ± 0.55	0.32 ± 0.55	1.01 ± 0.58	0.51 ± 0.34
3	NO	Alesund	ALES	1.70 ± 0.19	0.59 ± 0.19	0.64 ± 0.28	0.79 ± 0.35
4	NO	Heimsjø	HEMC	3.30 ± 0.91	0.56 ± 0.91	-0.82 ± 1.00	-1.32 ± 0.35
5	NO	Bodo	BODS	4.20 ± 0.31	1.41 ± 0.31	-1.61 ± 0.49	-1.02 ± 0.48
6	NO	Harstad	BJAC	2.30 ± 0.45	0.89 ± 0.45	-0.17 ± 0.50	-0.97 ± 0.45
7	NO	Tromsø	TROM	2.70 ± 0.29	0.64 ± 0.29	-0.17 ± 0.38	-0.07 ± 0.33

References

A, G., J. Wahr, and S. Zhong (2013), Computations of the viscoelastic response of a 3-d compressible earth to surface loading: an application to glacial isostatic adjustment in antarctica and canada, *Geophysical Journal International*, *192*(2), 557–572, doi:093/gji/ggs030.

Bos, M. S., R. M. S. Fernandes, S. D. P. Williams, and L. Bastos (2013), Fast error analysis of continuous gnss observations with missing data, *Journal of Geodesy*, *87*(4), 351–360, doi:007/s00190-012-0605-0.

Bos, M. S., S. D. P. Williams, I. B. Araújo, and L. Bastos (2014), The effect of temporal correlated noise on the sea level rate and acceleration uncertainty, *Geophysical Journal International*, *196*, 1423–1430, doi:10.1093/gji/ggt481.

Chao, B. F., Y. H. Wu, and Y. S. Li (2008), Impact of artificial reservoir water impoundment on global sea level., *Science*, *320*(5873), 212–4, doi:10.1126/science.1154580.

Cheng, M., B. D. Tapley, and J. C. Ries (2013), Deceleration in the earth's oblateness, *Journal of Geophysical Research: Solid Earth, Volume 118, Issue 2, pp. 740-747*, *118*(2), 740–747, doi:doi:10.1002/jgrb.50058.

Clark, J. A. (1977), Future sea-level changes due to west antarctic ice sheet fluctuations, *Nature*, *269*, 206–209, doi:10.1038/269206a0.

Dobslaw, H., F. Flechtner, I. Bergmann-Wolf, C. Dahle, R. Dill, S. Esselborn, I. Sasgen, and M. Thomas (2013), Simulating high-frequency atmosphere-ocean mass variability for dealiasing of satellite gravity observations: Aod1b rl05, *Journal of Geophysical Research:*

Oceans, Volume 118, Issue 7, pp. 3704-3711, *118*(7), 3704–3711, doi:10.1002/jgrc.20271.

Good, S. A., M. J. Martin, and N. A. Rayner (2013), EN4: Quality controlled ocean temperature and salinity profiles and monthly objective analyses with uncertainty estimates, *Journal Of Geophysical Research-Oceans*, *118*(12), 6704–6716, doi:002/2013JC009067.

Gouretski, V., and F. Reseghetti (2010), On depth and temperature biases in bathythermograph data: Development of a new correction scheme based on analysis of a global ocean database, *Deep-Sea Research Part I-Oceanographic Research Papers*, *57*(6), 812–833, doi:016/j.dsr.2010.03.011.

Kierulf, H. P., H. Steffen, M. J. R. Simpson, M. Lidberg, P. Wu, and H. Wang (2014), A gps velocity field for fennoscandia and a consistent comparison to glacial isostatic adjustment models, *Journal Of Geophysical Research: Solid Earth*, *119*(8), 6613–6629, doi:002/2013JB010889.

Klees, R., E. A. Revtova, B. C. Gunter, P. Ditmar, E. Oudman, H. C. Winsemius, and H. H. G. Savenije (2008), The design of an optimal filter for monthly grace gravity models, *Geophysical Journal International*, *175*(2), 417–432, doi:111/j.1365-246X.2008.03922.x.

Klinger, B., T. Mayer-Gürr, S. Behzadpour, M. Ellmer, A. Kvas, and N. Zehentner (2016), The new itsg-grace2016 release, *EGU General Assembly 2016, Vienna, Austria*, doi:10.13140/RG.2.1.1856.7280.

Lehner, B., C. R. Liermann, C. Revenga, C. Vörösmarty, B. Fekete, P. Crouzet, P. Döll, M. Endejan, K. Frenken, J. Magome, et al. (2011), High-resolution mapping of the world's reservoirs and dams for sustainable river-flow management, *Frontiers in Ecology and the Environment*, *9*(9), 494–502, doi:10.1890/100125.

Marzeion, B., P. W. Leclercq, J. G. Cogley, and A. H. Jarosch (2015), Brief communication: Global reconstructions of glacier mass change during the 20th century are consistent, *The Cryosphere*, *9*(6), 2399–2404, doi:194/tc-9-2399-2015.

Milne, G. A., and J. X. Mitrovica (1996), Postglacial sea-level change on a rotating earth: first results from a gravitationally self-consistent sea-level equation, *Geophysical Journal, Volume 126, Issue 3, pp. F13-F20.*, *126*(1), F13–F20, doi:10.1111/j.1365-246X.1996.tb04691.x.

Noël, B., W. J. van de Berg, E. van Meijgaard, P. Kuipers Munneke, R. S. W. van de Wal, and M. R. van den Broeke (2015), Evaluation of the updated regional climate model racmo2.3: summer snowfall impact on the greenland ice sheet, *The Cryosphere*, *9*(5), 1831–1844, doi:10.5194/tc-9-1831-2015.

Pawlowicz, R., T. McDougall, R. Feistel, and R. Tailleux (2012), An historical perspective on the development of the thermodynamic equation of seawater - 2010, *Ocean Science*, *8*, 161–174, doi:10.5194/os-8-161-2012.

Peltier, W. R., D. F. Argus, and R. Drummond (2015), Space geodesy constrains ice age terminal deglaciation: The global ICE-6G C (VM5a) model, *Journal Of Geophysical Research: Solid Earth*, *120*(1), 450–487, doi:002/2014JB011176.

Pfeffer, W. T., A. A. Arendt, A. Bliss, T. Bolch, J. G. Cogley, A. S. Gardner, J.-O. Hagen, R. Hock, G. Kaser, C. Kienholz, E. S. Miles, G. Moholdt, N. Mölg, F. Paul, V. Radic, P. Rastner, B. H. Raup, J. Rich, and M. J. Sharp (2014), The randolph glacier inventory: a globally complete inventory of glaciers, *Journal of Glaciology*, *60*(221), 537–552, doi:10.3189/2014JoG13J176.

Proudman, J. (1960), The condition that a long-period tide shall follow the equilibrium-law, *Geophysical Journal International*, *3*(2), 244–249, doi:10.1111/j.1365-246X.1960.tb00392.x.

Rignot, E., I. Velicogna, M. R. van den Broeke, A. Monaghan, and J. T. M. Lenaerts (2011), Acceleration of the contribution of the greenland and antarctic ice sheets to sea level rise, *Geophysical Research Letters*, *38*(5), L05,503, doi:10.1029/2011GL046583.

Roemmich, D., and J. Gilson (2009), The 2004-2008 mean and annual cycle of temperature, salinity, and steric height in the global ocean from the argo program, *Progress in Oceanography, Volume 82, Issue 2, p. 81-100.*, *82*(2), 81–100, doi:10.1016/j.pocean.2009.03.004.

Shepherd, A., E. R. Ivins, G. A, V. R. Barletta, M. J. Bentley, S. Bettadpur, K. H. Briggs, D. H. Bromwich, R. Forsberg, N. Galin, M. Horwath, S. Jacobs, I. Joughin, M. A. King, J. T. M. Lenaerts, J. Li, S. R. M. Ligtenberg, A. Luckman, S. B. Luthcke, M. McMillan, R. Meister, G. Milne, J. Mouginot, A. Muir, J. P. Nicolas, J. Paden, A. J. Payne, H. Pritchard, E. Rignot, H. Rott, L. S. Sørensen, T. A. Scambos, B. Scheuchl, E. J. O. Schrama, B. Smith, A. V. Sundal, J. H. van Angelen, W. J. van de Berg, M. R. van den Broeke, D. G. Vaughan, I. Velicogna, J. Wahr, P. L. Whitehouse, D. J. Wingham, D. Yi, D. Young, and H. J. Zwally (2012), A reconciled estimate of ice-sheet mass balance., *Science*, *338*(6111), 1183–9, doi: 10.1126/science.1228102.

Swenson, S., D. Chambers, and J. Wahr (2008), Estimating geocenter variations from a combination of grace and ocean model output, *Journal Of Geophysical Research-Solid Earth*, *113*(B8), doi:029/2007JB005338.

Tamisiea, M. E., E. M. Hill, R. M. Ponte, J. L. Davis, I. Velicogna, and N. T. Vinogradova (2010), Impact of self-attraction and loading on the annual cycle in sea level, *Journal of Geophysical Research: Oceans*, *115*(C7), C07,004, doi:10.1029/2009JC005687.

van den Broeke, M. R., E. M. Enderlin, I. M. Howat, P. Kuipers Munneke, B. P. Y. Noël, W. J. van de Berg, E. van Meijgaard, and B. Wouters (2016), On the recent contribution of the greenland ice sheet to sea level change, *The Cryosphere*, *10*(5), 1933–1946, doi:10.5194/tc-10-1933-2016.

van Wessem, J. M., C. H. Reijmer, M. Morlighem, J. Mouginot, E. Rignot, B. Medley, I. Joughin, B. Wouters, M. A. Depoorter, J. L. Bamber, J. T. M. Lenaerts, W. J. van de Berg, M. R. van den Broeke, and E. van Meijgaard (2014), Improved representation of east antarctic surface mass balance in a regional atmospheric climate model, *Journal of Glaciology*, *60*(222), 761–770, doi:10.3189/2014JoG14J051.

Wada, Y., L. P. H. van Beek, C. M. van Kempen, J. W. T. M. Reckman, S. Vasak, and M. F. P. Bierkens (2010), Global depletion of groundwater resources, *Geophysical Research Letters*, *37*(20), doi:029/2010GL044571.

Wada, Y., D. Wisser, and M. F. P. Bierkens (2014), Global modeling of withdrawal, allocation and consumptive use of surface water and groundwater resources, *Earth System Dynamics*, *5*(1), 15–40, doi:194/esd-5-15-2014.

Watkins, M. M., D. N. Wiese, D.-N. Yuan, C. Boening, and F. W. Landerer (2015), Improved methods for observing earth's time variable mass distribution with grace using spherical cap mascons, *Journal of Geophysical Research: Solid Earth*, *120*(4), 2648–2671, doi: 10.1002/2014JB011547.

Whitehouse, P. L., M. J. Bentley, G. A. Milne, M. A. King, and I. D. Thomas (2012), A new glacial isostatic adjustment model for antarctica: calibrated and tested using observations of relative sea-level change and present-day uplift rates, *Geophysical Journal International*, *190*(3), 1464–1482, doi:111/j.1365-246X.2012.05557.x.

Woodworth, P. L. (2012), A note on the nodal tide in sea level records, *Journal Of Coastal Research*, *28*(2), 316–323, doi:112/JCOASTRES-D-11A-00023.1.
